# Incidental ^18^F-FDG Uptake of the Pubic Ramus and Abdominal Muscles due to Athletic Pubalgia During Acute Prostatitis

**DOI:** 10.4274/mirt.19484

**Published:** 2018-10-09

**Authors:** Olivier Rager, Marlise Picarra, Emmanouil Astrinakis, Valentina Garibotto, Gaël Amzalag

**Affiliations:** 1 University Hospital of Geneva, Clinic of Nuclear Medicine, Geneva, Switzerland; 2 University Hospital of Geneva, Clinic of Radiology, Geneva, Switzerland

**Keywords:** PET/CT, magnetic resonance imaging, athletic pubalgia, sports hernia, prostatitis

## Abstract

A 23-year-old African native male patient presented with fever, lumbalgia and dysuria after returning from a trip to Togo. His physical examination revealed pain over the pubic symphysis and rectal tenderness on digital exam. The C-reactive protein (CRP) level was elevated along with positive blood and urinary cultures for methicillin-resistant Staphylococcus aureus. An magnetic resonance imaging that has been performed to rule out arthritis/osteomyelitis in the pubis revealed edema of the symphysis. An ^18^F-FDG positron emission tomography/computed tomography supported the diagnosis of prostate infection and showed a focal uptake of the pubic symphysis, with diffuse hyper-metabolism of the insertions of the rectus abdominis and longus adductor muscles, corresponding to athletic pubalgia. Fever and CRP responded rapidly to antibiotherapy.

## Abstract (tr)

Yirmi üç yaşında Afrikalı erkek hasta Togo seyahati sonrası ateş, lumbalji ve disüri şikayetleri ile başvurdu. Fizik muayenede symphysis pubis üzeri ağrı ve rektal tuşede hassasiyet mevcuttu. C-reaktif protein (CRP) düzeyi yüksekti ve kan ve idrar kültürlerinde metisiline dirençli Staphylococcus aureus üremesi saptandı. Pubiste olası bir artrit/osteomiyeliti ekarte etmek için istenen manyetik rezonans görüntülemede symphysiste ödem görüldü. ^18^F-FDG pozitron emisyon tomografisi/bilgisayarlı tomografi prostat enfeksiyonu tanısını doğruladı. Bununla birlikte atletik pubaljiyle uyumlu olarak symphysis pubiste fokal tutulum ve rektus abdominis ve adduktor longus kas insersiyolarında diffüz hipermetabolizm görüldü. Antibiyoterapi sonrası ateş ve CRP düzeyi hızla düzeldi.

## Figures and Tables

**Figure 1:**
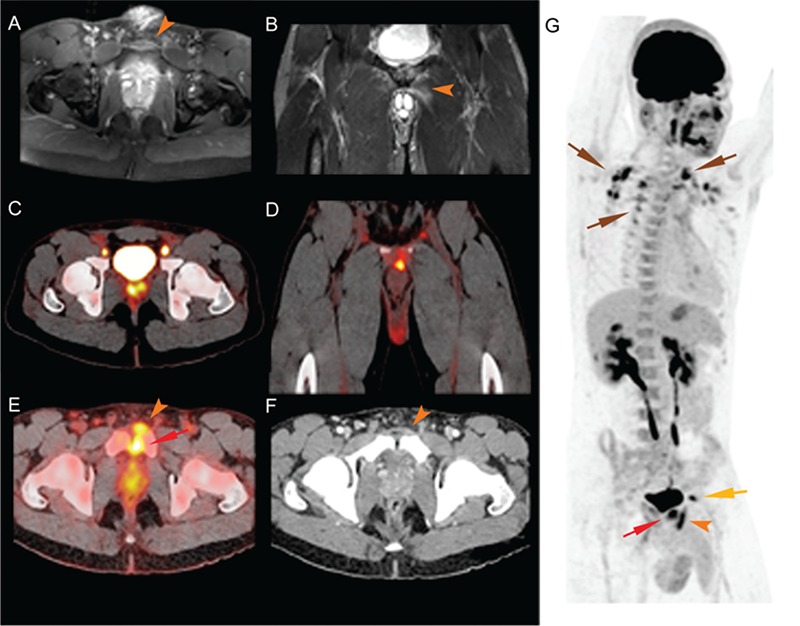
We present the case of a 23-year-old male African native patient presenting with fever, lumbalgia and dysuria after returning from a trip to Togo. The patient is a professional athlete (soccer player) with a known history of malaria during childhood. On palpation, there was pain over the pubic tubercle and the digital rectal exam was tender and sensitive. The blood formula was normal except elevated [C-reactive protein (CRP): 118 mg/L]. Recurrence of malaria had been excluded by repeated thick blood smears. Both blood and urinary cultures were positive for methicillinresistant *Staphylococcus aureus*. Computed tomography (CT) with nephrographic contrast and dedicated ultrasound ruled out pyelonephritis. A pelvic magnetic resonance imaging (MRI) was performed to rule out arthritis and osteomyelitis that revealed a thickening of the aponeurosis of the left rectus abdominis muscle on T1-weighted axial sequence after injection of gadolinium (A, arrow head), a hyper-signal of the symphysis on the STIR-weighted sequence corresponding to marrow edema without articular effusion, and a hyper-signal corresponding to a strain of the left adductor longus muscle (B, arrow head) characteristic of athletic pubalgia (1,2). ^18^F-FDG positron emission tomography/CT (PET/CT) found an increased prostatic tracer uptake along with bilateral external iliac lymph nodes hyper-metabolism (C), and also showed hyper-metabolism of the insertion of the left longus adductor (D) and of the left rectus abdominis (E and G, orange arrow) with a focal uptake in the pubic symphysis (E and G, red arrow) that were in concordance with the MRI findings. Increased ^18^F-FDG uptake on the molecular inversion probe sequence (G) in the supraclavicular, latero-cervical and para-vertebral regions corresponded to activated brown adipose tissue (brown arrow), the yellow arrow corresponds to the left iliac node; the right iliac node and the prostate were masked by the bladder. The CT scan (F) with contrast media confirmed the findings (thickening of the aponeurosis of the left rectus abdominis, arrow head). A prostatic origin of the infection was presumed and antibiotic therapy was initiated (intravenous vancomycin, then co-trimoxazole per os). Regression of fever, normalization of CRP and clearing of the cultures were observed rapidly. Sports hernia/athletic pubalgia is an activity-related lower abdominal and proximal adductor-related pain seen in athletes (3,4,5,6). Symptoms are most often unilateral but are not uncommonly bilateral. This pattern with hyper-metabolism of the insertion of the muscles associated with uptake in the pubic symphysis due to inflammation should be recognized on imaging not to be mistaken for a muscle abscess (7,8). To the best of our knowledge, this specific feature in ^18^F-FDG PET/CT had not been previously described in the literature.
